# Introduction of a guide based on a femoral neck section for fixation with multiple screws: a cadaveric study

**DOI:** 10.1186/s12891-018-2026-6

**Published:** 2018-04-04

**Authors:** Qiuliang Zhu, Bin Xu, Jinzhu Lv, Maohua Yan

**Affiliations:** Department of Orthopedics, People’s Hospital of Anji, Zhejiang, 313000 China

**Keywords:** Femoral neck fracture, Internal fixation, Guide, Multiple screws, Femoral neck section

## Abstract

**Background:**

The design and application of assisted instrumentation for internal fixation of femoral neck fractures with multiple screws are still evolving. A novel guide based on a femoral neck section was designed to improve the accuracy of screw placement, and its efficacy was evaluated.

**Methods:**

A guide based on a femoral neck section was designed for assisted fixation of femoral neck fractures with multiple screws. Femoral specimens from 10 adults (20 femurs) underwent assisted internal fixation for a femoral neck fracture with 3 cannulated screws using the new guide technique or conventional technique. The accuracy of screw orientation and entry point, the accuracy of optimal screw positioning, and drilling attempts, operative time, and fluoroscopy time were recorded.

**Results:**

Among all 20 specimens, 60 screws were inserted successfully. Screw parallelism, operative time, and fluoroscopy time showed no statistical difference between the new guide technique and conventional technique (*P* > 0.05). The accuracy of optimal screw positioning was determined by the contained screw area ratio, distance between screws, distance from the centre of the femoral neck section, distance between screws and the femoral neck cortex, and Drilling attempts were statistical significantly better (data in the first three were larger and in the latter two was smaller) with the new guide technique, than with conventional technique (*P* < 0.05).

**Conclusions:**

This new, two-dimensional, fluoroscopy-assisted, percutaneous guide technique enables accurate and optimal screw positioning in internal fixation of femoral neck fractures, compared with conventional technique.

## Background

Use of 3 cannulated screws has been the standard method for internal fixation of femoral neck fractures in young patients and elder adults with nondisplaced fractures. Bhandari et al. [1] queried 442 surgeons and found that 82.8% preferred cannulated screws for nondisplaced fractures, with a 17% preference for displaced fractures. Luttrell et al. [2] surveyed 272 Orthopaedic Trauma Association members regarding treatment of high-angle “vertical” femoral neck fractures, and found that 43.1% preferred cannulated screws, with or without off-axis screw placement. Some thought that optimal biomechanical stability and minimally-invasive technique could be achieved if accuracy of optimal screw placement could be maintained.

For accuracy of optimal screw placement, two-dimensional, fluoroscopy-assisted commercial guides and improved versions have focused on screw orientation and entry point. The advantage was reflected in reduction of operative and fluoroscopy time [3–5]. Three-dimensional, computer-assisted navigation systems generated more discussion on the accuracy of optimal screw positioning. Related studies showed that navigation systems were superior for parallel screw placement, had greater spread, and distributed cortical support using a femoral neck section (FNS), but the complex equipment, complicated surgical procedure, and prolonged operative time limited clinical use [6–9]. Therefore, the design and application of assisted instrumentation for internal fixation of femoral neck fractures with multiple screws are still evolving.

We designed a novel instrument based on the specific anatomy of the FNS for internal fixation of femoral neck fractures, and called it “the guide of femoral neck section (GFNS).” The present study demonstrated that simulated surgery using GFNS for internal fixation of femoral neck fractures enhanced accuracy of screw position in 10 dry cadaveric femurs under two-dimensional fluoroscopic (C-arm) control, compared with conventional technique.

## Methods

**Fig. 1:**
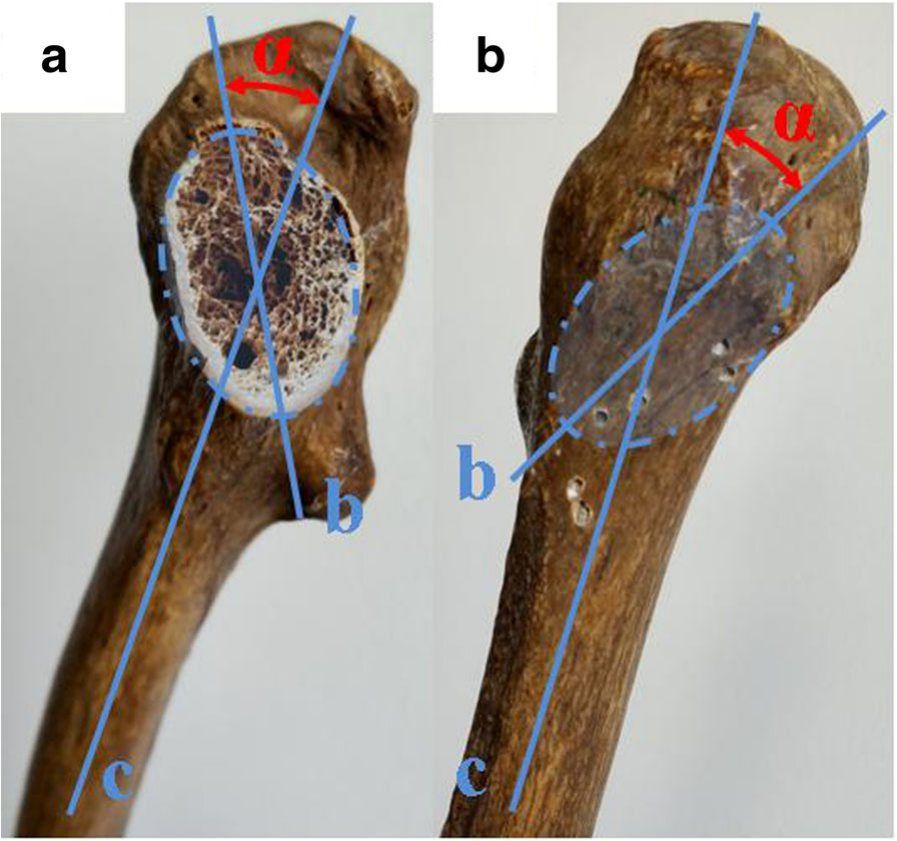
Picture showed that the shape of femoral neck section (FNS) related with the medial aspect (A) and lateral surface (B) of proximal femur: The ellipse represented FNS; ∠α Femoral neck torsion angle; b Long axis of FNS; c Coronal axis of proximal femur

The shape of FNS (Fig. 1): FNS was roughly elliptical. The long axis of the ellipse rotated forward formed an angle with the coronal plane of the proximal femur called the femoral neck torsion angle (FNTA), with a value of approximately 20° [10–12]. Thus, iatrogenic perforation could occur in the posterosuperior and anterorinferior quadrants of the lateral femoral wall during internal fixation of the femoral neck with multiple screw insertions [13].

**Fig. 2:**
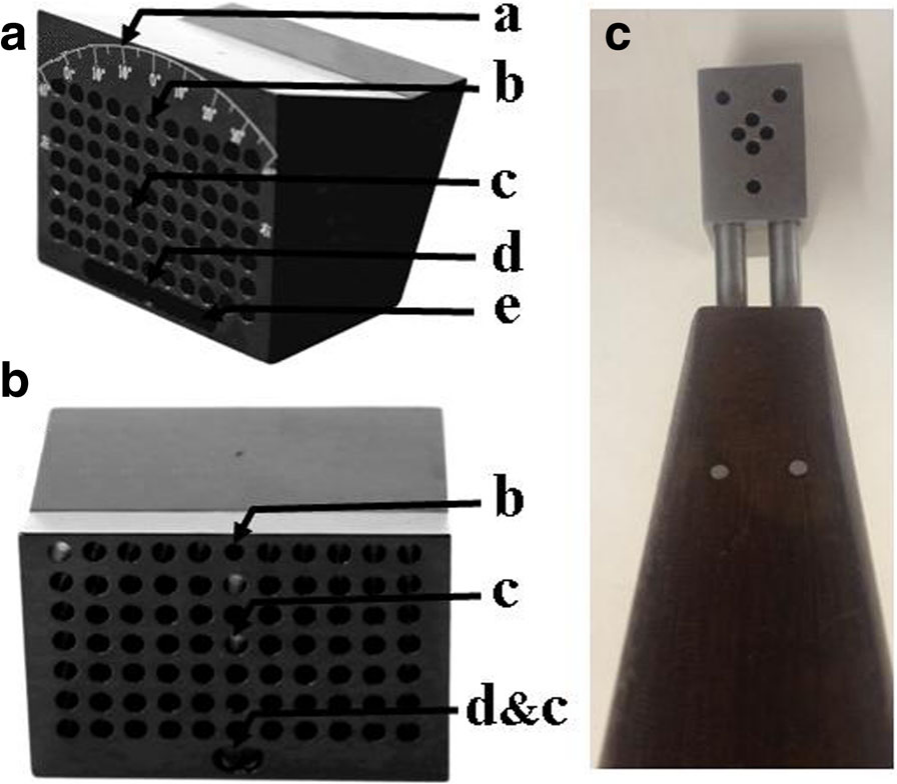
Overall view (A) and medial surface (B) of the guide of femoral neck section (GFNS) and the conventional guide (C) supplied by manufacturer for internal fixation of femoral neck fracture: a angle logo; b mounting hole; c parallel guide hole; d centre hole; e adjust hole

GFNS (Fig. 2): GFNS is made of titanium, making it lighter (about 220 g) and easier to fix, and consists of a carrier with angle logo and a cuboid. The cuboid is 6-cm long, with 8 rows and 11 columns of parallel guide holes, each with diameter of 2.6 mm, with 2.4-mm hole spacing. The bottom row includes a centre hole and adjustment holes, for connection with the single hole at the inner surface of the cuboid, with hole spacing of 2.1 mm. At the top of the lateral aspect of the cuboid, the angle logo carrier is set on an angle with the bottom row centre hole at the centre of acircle 0 °- 40 ° to the left and right, matching the FNTA. Anon-parallel mounting hole with 2 mm diameter is below the logo. The elevation angle of the inner surface of the cuboid is 130°, matching the surface soft tissue of the thigh.

Specimens: 20 dry, intact femoral specimens from 10 adults (7 men and 3 women; average age 47.5 years, range, 37 to 58y) were obtained from the Department of Anatomy, Zhejiang University School of Medicine. No visible deformity and no defects on distal and proximal femurs were observed. Two different operating techniques were used in the right or left femur of one adult, and were grouped using random numbers. The long and short axis of the femoral neck and the FNTA were calculated (Table 1). All femurs were mounted on an operating table and covered with a surgical drape to imitate soft tissue for percutaneous screw placement.

**Table 1:** The general data of 20 femurs

Group	Number	Femoral neck section (mm)		Femoral neck torsion angle (deg.)
		Long axis	Short axis	
DFNS	10	36.67 ± 3.62*	25.57 ± 2.44*	19.30 ± 3.01*
Conventional	10	37.08 ± 2.74	24.98 ± 2.95	20.12 ± 2.36

*P > 0.05 Comparing with Conventional Group

### Technique description

Conventional AO technique: First, the guide wire was freehand inserted from the trochanteric lateral wall to the femoral head under image intensification, to ensure that the guide wire was located on the central axis of the femoral neck in anteroposterior (AP) and lateral views. Second, a manufacturing guide (Chuangsheng, Jiangsu, China. Figure 2C) was placed over the wire through its central hole, with selection of 3 appropriate outer holes for greater spread close to the femoral neck cortex, and an inverted triangle was constructed, followed by insertion of 3 guide-wires. The manufacturing guide and the central wire were removed, with verification of the correct placement of wires using C-arm fluoroscopy in AP and lateral views. Finally, 7.3-mm cannulated screws were inserted over the guide wires.

**Fig. 3:**
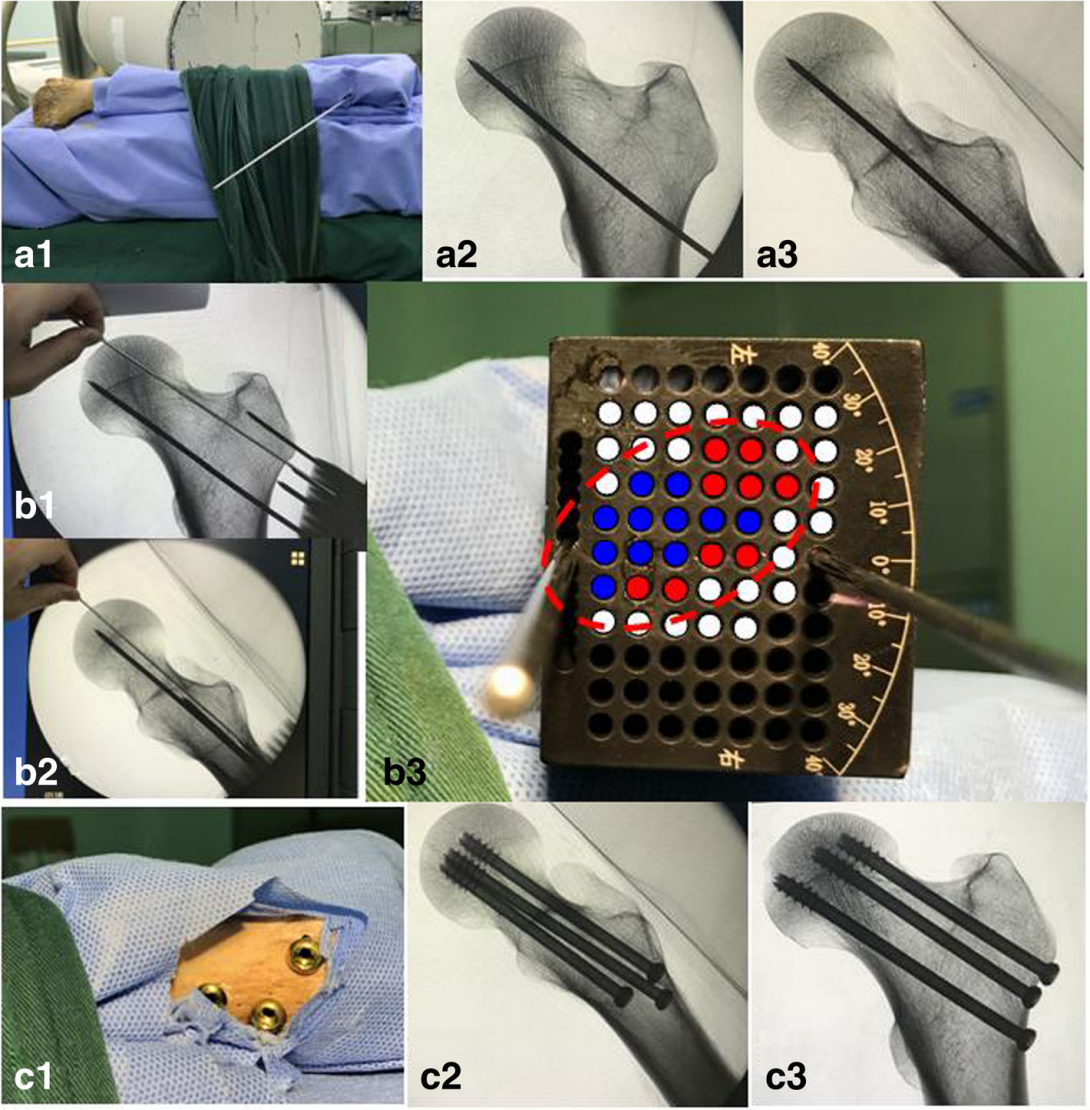
The operative process with GFNS: Inserted the first guide-wire into appropriate place with freehand under AP and lateral views (A). Fixed and draw FNS on GFNS (red ellipse) by related data, identify the excellent hole (red point), good hole (blue point) and bad hole (white hole). After making sure the guide wires were in ideal positions by lateral and AP image, fixed with appropriate length screws

GFNS technique (Fig. 3): With C-arm fluoroscopy guidance, the first wire (inferior wire) was placed freehand in the middle or slightly posterior region of the femoral neck on the lateral view, parallel to the femoral neck axis and attached to the femoral calcar on AP projection. GFNS was placed over the wire through the central hole on the bottom (wire anteversion could be adjusted using the adjustment holes on the bottom, if needed). A 2-mm Kirschner wire was inserted along the fixing hole to the greater trochanter to fix the GFNS. Assuming 20°placement relative to the pre-measured long axis of the femoral neck, 2-4 guide wires inserted into GFNS but outside the femoral cortex were adjusted using AP and lateral images. The edge of FNS was confirmed and the projection of FNS (red line) on GFNS was drawn. The screw positions were located such that hole placement was within 3 mm of the femoral neck cortex (considered excellent, red spot); location in the middle of the FNS is considered good (blue spot) and location outside the red line is poor (white spot). Using the “excellent” guide-holes, 3 were selected for maximum distance from each other. The second wire (anterosuperior) was inserted and placed near the anterosuperior cortex. The third guide wire (medial and posterior wire) was placed close to the medial and posterior cortex. After making sure the guide wires were in ideal positions using lateral and AP images, appropriate length screws were used for fixation.

### Measurement parameters

Screw accuracy of orientation and placement: Screw parallelism, distance between screws (defined as total length of connecting lines between 3screw centres), screw area ratio (consisting of the triangle screw area and the FNS area), distance from the centre of FNS to the screws, and distance from the screws to the femoral neck cortex were measured by a resident who was blinded to the surgical procedure.

Operative time was measured from the moment of initial wire placement to the point when the 3screws were finally fixed. Fluoroscopy time was determined by the number of fluoroscopic single shots during the operation. Perforation for insertion of a guide wire into the femoral trochanteric wall, including drill reversal, was counted as a drilling attempt.

### Statistical methods

Analyses were performed using SPSS version 16.0 statistical software (SPSS Inc., Chicago, IL, USA). Values are presented as mean ± SD. The paired Student’s t-test was used to compare the measured parameters between 2groups, and *p* values ≤0.05 were considered statistically significant.

## Results

Among all 20 cases, 60 screws were inserted successfully. The results of measurements of screw placement accuracy are presented in Table 2 and the parameters measured during surgery are shown in Table 3.

**Table 2:** Comparative analysis of screw placement accuracy in 2 groups

group	Screw parallelism (deg.)		Screw area ratio	DS (mm)	DC (mm)	DSC (mm)
	AP	Lateral				
conventional	1.73 ± 0.62	1.52 ± 0.60	0.16 ± 0.02	10.38 ± 3.91	8.02 ± 1.51	4.21 ± 1.31
GFNS	1.68 ± 0.83#	1.61 ± 0.57#	0.29 ± 0.02*	16.87 ± 4.12*	11.05 ± 1.94*	2.40 ± 1.24*

*GFNS* guide of femoral neck section, *DS* distance between screws, *DC* distance between the centre of FNS, *DSC* distance between screw to femoral neck cortex

*P < 0.05; #*p* > 0.05 Comparing with Conventional group

**Table 3:** comparative analysis of parameters during surgical procedure in 2 groups

Group	Operation time (min)	Fluoroscopic time (no.)	Drilling attempt (no.)
conventional	24.80 ± 9.12	8.89 ± 2.17	7.97 ± 3.60
GFNS	27.33 ± 7.61#	9.51 ± 1.72#	4.81 ± 1.27*

*P < 0.05; #*p* > 0.05 Comparing with Conventional group

Screw parallelism on AP and lateral imaging showed no statistical difference between the 2 groups. Screw area ratio, distance between screws, distance from the centre of FNS, and distance from screws to femoral neck cortex in GFNS group were statistical significantly better (data in the first three were larger and in the latter was smaller) than those in the conventional group (*P* < 0.05). The operative and fluoroscopy times showed no statistical difference between the 2 groups. Drilling attempts with GFNS were significantly fewer than with use of the conventional guide.

## Discussion

Despite superior biomechanical properties and greater fracture stability with sliding hip screws in internal fixation of femoral neck fractures, use of 3 cannulated screw has been the most popular technique, as superior torsion stability, minimally-invasive insertion, and limited disruption of femoral head blood supply may be lead to better postoperative function [1, 2, 14–16]. Greater screw spread, screw placement close to the cortex for cortical support, and parallel screw placement reflect screw placement accuracy, and are thought to achieve greater stability and decreased risk of nonunion of the fracture [17–20]. For accuracy of screw placement, a series of guides and instruments have been developed to assist the surgical procedure. However, there is no consensus regarding the optimal instrument or guide for both screw precision and minimum invasion.

Two-dimensional, fluoroscopy (C-arm)-assisted guides have been used to improve the technique of femoral neck fracture repair, with improved versions reported by Xia et al. and Yuenyongviwat et al. in synthetic femurs [3, 5]. These studies showed the benefits of reduced operative time and radiation exposure but did not consider screw position accuracy. Yin et al. and Tai et al. described a novel guide/screw (as a sleeve) that was simple in structure and easy to apply, and that facilitated accuracy of guide wire orientation and entry point. However, the accuracy of optimal screw distribution in FNS has not been considered [4, 21]. Three-dimensional, computer-assisted navigation systems have been increasingly studied [6–9]. Previous literature demonstrated that the accuracy of screw placement distribution, which included the guide wire entry point, orientation, and optimal screw distribution, was improved with use of navigation systems, but the radiation exposure and operative time were increased. In addition, the complex equipment and complicated surgical procedure (scanning, registering, planning, etc.) limited the use in clinical practice.

The advantage of the accuracy associated with screw placement distribution in femoral neck fixation was demonstrated in the present study. This is because the projection of FNS is clearly drawn on the GFNS using the following process: with reference to the first guide wire placement, based on previous measured data (FNTA, long and short axis of FNS) or historical information, the AP and lateral intraoperative images were integrated to form the real-time shape of FNS. Therefore, according to the requirement of optimal screw position for greater spread and cortical support, the “excellent” guide-holes were easily confirmed on visible projection (Fig. 3B3). In this study, the screw area ratio, distance between screws, and distance from the centre of FNS were greater in GFNS compared with those in the conventional group, and the distance from the screws to the femoral neck cortex was smaller. The results were better than with use of two-dimensional, fluoroscopy-assisted guide technique and similar three-dimensional, computer-assisted systems described in previous literature [7, 8]. The improvement in accuracy of screw orientation and entry point were not considered in the design of GFNS; as a result, the screw parallelism showed no significant difference between the 2 groups in this study.

This study used fewer drilling attempts with GFNS technique than with conventional technique. Multiple drilling attempts might weaken the bone and lead to unsuccessful adjustment, as the guide wire may slide along the initial drilling track. With GFNS technique, before guide wire insertion, a virtual extended drilling track from the “excellent” hole was confirmed on fluoroscopy (Fig. 3), limiting the need for further drilling attempts. The results are analogous to those in previous reports with computer-assisted navigation technique [7, 8]. The reduction in drilling attempts shortened the operative and fluoroscopy time, but the time needed to verify the “excellent” drilling holes on GFNS was prolonged; therefore, the operative and fluoroscopy times were similar in the GFNS group and conventional groups.

The limitation of this study is the inadequacy of soft tissue coverage and femoral neck fracture simulation models. In clinical practice, thicker soft tissue coverage may interfere with the accuracy of screw orientation and entry point. Use of a surgical drape to simulate thin soft tissue probably oversimplified the complexity of the surgical procedure, suggesting that the percutaneous GFNS technique should not be used in obese patients. To simplify the trial process, we used the intact proximal femur to simulate anatomic reduction of a femoral neck fracture. This may have decreased the difficulty of the surgical procedure, but had little influence on the trial results.

## Conclusion

Two-dimensional, fluoroscopy-assisted, percutaneous GFNS technique can increase the accuracy of optimal screw positioning and can decrease drilling attempts in internal fixation of femoral neck fractures. There was no difference in accuracy of screw orientation and entry point or operative and fluoroscopy time compared with conventional technique. The percutaneous GFNS technique should be considered for use in clinical practice, as three-dimensional, computer-assisted navigation systems have not been widely employed.

## Abbreviations

AP: Anteroposterior view
DC: Distance between the centre of FNS
DS: Distance between screws
DSC: Distance between screw to femoral neck cortex
FNS: Femoral neck section
FNTA: Femoral neck torsion angle
GFNS: Guide of femoral neck section
